# Development and validation of a novel pre-operative comprehensive prognostic score in esophageal squamous cell carcinoma

**DOI:** 10.17305/bjbms.2021.6350

**Published:** 2021-10-19

**Authors:** Jifeng Feng, Liang Wang, Xun Yang, Qixun Chen

**Affiliations:** Department of Thoracic Oncological Surgery, Institute of Cancer Research and Basic Medical Sciences of Chinese Academy of Sciences, Cancer Hospital of University of Chinese Academy of Sciences, Zhejiang Cancer Hospital, Hangzhou, China

**Keywords:** Prognostic score, esophageal squamous cell carcinoma, squamous cell carcinoma antigen, C-reactive protein to albumin ratio, neutrophil to lymphocyte ratio, fibrinogen, cancer-specific survival

## Abstract

We herein propose a novel integrative score based on inflammatory and nutritional score, coagulation indicator and tumor marker, named comprehensive prognostic score (CPS), to predict post-operative survival in resectable esophageal squamous cell carcinoma (ESCC). We also aimed to establish and validate a nomogram based on CPS and other clinical features for individual survival prediction. A total of 490 resectable ESCC patients were randomly divided into either a training or validation cohort at a ratio of 7:3 for retrospective analysis. The CPS, based on squamous cell carcinoma antigen, C-reactive protein to albumin ratio, neutrophil to lymphocyte ratio, and fibrinogen, was divided into two models to verify its prognostic value. The predictive model of CPS-based nomogram was established and validated in two cohorts. The patients with CPS low group in Model 1 had better 5-year cancer-specific survival (CSS) than those in CPS high group (50.7% vs. 17.8%, *p* < 0.001). For Model 2, the 5-year CSS for CPS 0, 1 and 2 were 75.0%, 38.9% and 13.3%, respectively (*p* < 0.001). CPS was confirmed as an independent prognostic score in both models. The CPS-based nomogram can accurately and effectively predict survival in resected ESCC. The CPS is a novel, simple, and effective predictor in resectable ESCC. Moreover, CPS has a potential independent prognostic value in predicting post-operative survival, which can accurately and effectively predict individual survival in resectable ESCC.

## INTRODUCTION

Esophageal cancer (EC) is one of the most common aggressive malignancies [1]. According to the 2018 Global cancer statistics, a total of 18.1 million new cases (0.57 million for EC) were diagnosed and 9.6 million cases (0.51 million for EC) died from cancer [1]. Squamous cell carcinoma (SCC) of the esophagus is one of the main types of EC, accounting for the vast majority in China and other highest-risk area of so-called “Asian EC Belt” [2,3]. Despite the improvements in treatment in recent years, the poor prognosis of esophageal squamous cell carcinoma (ESCC) highlights the need to refine more sensitive prediction methods, which are essential prior to treatment [4]. Therefore, exploring more novel prognostic scores in ESCC is still an important task.

Although the sensitivity and specificity of serum tumor markers are not high, they play an important significance in cancer diagnosis and prognosis. To improve the early diagnosis and prolong the survival time for cancer patients, it is necessary to explore various sensitive tumor markers in cancers. Serum SCC antigen (SCCA) is a commonly used clinical tumor marker for SCC, such as cervical SCC, head and neck SCC, and ESCC [5-7]. To date, however, the prognostic role of SCCA in patients with ESCC remains controversial [8,9].

It is well known that nutritional and inflammatory status is associated with cancer prognosis. Therefore, more and more inflammatory and/or nutritional predictors, such as C-reactive protein (CRP), neutrophil to lymphocyte ratio (NLR), albumin (ALB), and CRP to ALB ratio (CAR), have been applied either alone or in combination to various cancers in recent years [10-12]. However, these inflammatory and/or nutritional indicators mentioned above may be influenced by various factors, which are to some extent deficient.

As an important protein regarding coagulation, fibrinogen (FIB) is involved in the maintenance of hemostasis. Recent study reported that serum plasma FIB is considered as one of several acute phase reactant proteins in response to systemic inflammation or tissue injury [13]. Accumulating evidence has revealed that serum FIB was associated with progression and prognosis in a variety of cancers, while its role in ESCC is still controversial [14-16].

Nutritional and/or inflammatory status, coagulation-related indicators, and tumor markers may be influenced by a variety of non-cancer related conditions, which may lead to biased results. We hypothesized that the combination of these indicators could reduce the potential bias and improve the prognostic value. Therefore, we initially proposed a novel prognostic score, named (comprehensive prognostic score [CPS], based on a composite variable of SCCA, NLR, CAR and FIB), for predicting cancer-specific survival (CSS) in resectable ESCC patients. Moreover, comparisons of prognostic values between CPS and other conventional prognostic scores including systemic immune-inflammation index (SII), Glasgow prognostic score (GPS), and prognostic nutritional index (PNI) were also analyzed. Finally, a predictive nomogram regarding resected ESCC patients based on CPS was also constructed and validated to predict individual survival.

## MATERIALS AND METHODS

### Patient selection

**FIGURE 1:**
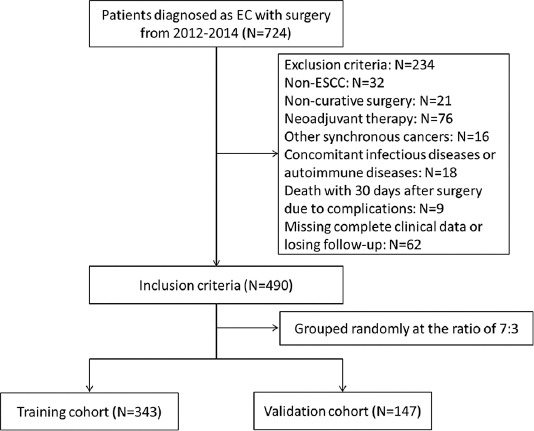
The flow diagram of selection of eligible patients. According to the inclusion and exclusion criteria, 490 patients were randomly divided into either a training (n=343) or validation cohort (n=147) at a ratio of 7:3 for analysis

The current research was a retrospective study including 490 ESCC cases with radical resection (R0) with the McKeown or Ivor Lewis procedure in our department from Jan. 2012 to Jun. 2014. The 7^th^ AJCC/UICC pathological TNM staging system including primary tumor (T), lymph node metastasis (N), and distant metastasis (M) were used for the current study [17]. All patients were randomly assigned to a training cohort (n = 343) or validation cohort (n = 147) at a ratio of 7:3. The inclusion and exclusion criteria were shown in Figure 1. Patients with pre-operative neoadjuvant treatment were excluded because neoadjuvant treatment might affect the hematological indicators. Post-operative adjuvant treatment is still uncertain. For ESCC patients with R0 resection, the NCCN guidelines only recommend regular follow-up. Therefore, not all patients in China received post-operative adjuvant therapy, which is mainly based on the surgeon’s recommendation and the physical and financial status of each patient [18,19]. Post-operative adjuvant treatments including cisplatin-based chemotherapy and/or radiotherapy with a median irradiation dose of 50 Gy (range: 40-56 Gy), but not mandatory, were conducted on the basis of the post-operative pathologic results with T3-T4 tumors and those with positive node metastasis [20,21]. The patients were followed up with regular checks. The last follow-up was completed in Dec. 2019.

### Data collection and CPS definition

The laboratory results, such as SCCA, lymphocyte, neutrophil, platelet, ALB, CRP and FIB, were obtained within 1 week before surgery. The definitions of CAR, NLR, PNI, GPS and SII refer to the previous studies [22,23]. The CPS was composed of four serum variables (SCCA, CAR, NLR and FIB). Two models were used for the current study. The model 1, as a continuous variable, was calculated according to the logistic equation by combining SCCA, NLR, CAR and FIB. The optimal cut-off point for CPS in model 1 then was plotted according to the receiver operating characteristic (ROC) curve. The model 2, as a categorical variable, each indicator was assigned to a score of 0 or 1 according to the optimal cut-off points for SCCA, NLR, CAR and FIB. The CPS in model 2 then was calculated as the summed score of 0 or 1, which was divided into 3 groups (CPS 0, 1 and 2, respectively).

### Ethical statement

All data including in the current study was anonymous and retrospective, informed consent was waived and the protocol was approved by the ethics committee (IRB.2021-5). The present study was conducted in accordance with the Declaration of Helsinki.

### Statistical analysis

Medcalc 17.6 (MedCalc Software bvba, Ostend, Belgium), R software (version 3.6.1, Vienna, Austria), and SPSS 20.0 (SPSS Inc., Chicago, IL, USA) were used to perform all statistical analyses in the current study. Variables in continuous or categorical were compared by the student’s *t*-test or Chi-square test or fisher’s exact test, respectively. ROC curves were carried out to select the optimal cut-off points for continuous variables. Univariate and multivariate analyses were performed and expressed as hazard ratios (HRs) and 95% confidence intervals. The areas under the curve (AUC) between CPS and other variables were compared. A prognostic nomogram was built based on the results in multivariate analyses. Calibrations for survival prediction were performed by comparing the two cohorts. Time-dependent ROC curves and decision curves were performed to evaluate the discriminative ability and predictive accuracy between nomogram and TNM. All statistical tests were two-sided and a *p* < 0.05 were considered to be statistically significant.

## RESULTS

### Patient characteristics in two cohorts

In the training cohort, the median age of enrolled patients in the current study was 58 years (range: 36-78 years), and the median follow-up time was 41 months (range: 5-94) months. There were 247 males (72.0%) and 96 females (28.0%). There were 98 males (66.7%) and 49 females (33.3%) with the mean age of 58.3 ± 8.0 years (range: 36-78 years) in the validation cohort. The mean values of SCCA, NLR, CAR and FIB were 0.98 ± 0.54 mg/L, 3.03 ± 1.24, 1.83 ± 2.28 and 3.80 ± 0.88 g/L in training set and 0.95 ± 0.48 mg/L, 3.21 ± 0.69, 1.81 ± 2.68 and 3.82 ± 0.82 g/L in validation set, respectively. The baseline characteristics between the cohorts were shown in Table 1. The tumor lengths in patients in the training cohort were larger than those in the validation cohort (4.3±1.8 vs. 3.9±1.9, *p* = 0.040). Otherwise, there was no significant difference between the two groups.

**TABLE 1:**
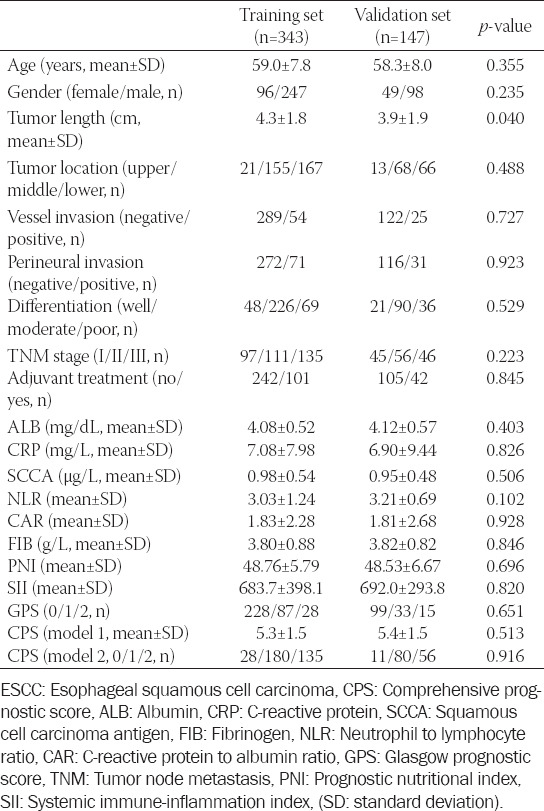
Baseline characteristics of ESCC patients in the training and validation sets

### Determination of CPS in two models

**FIGURE 2:**
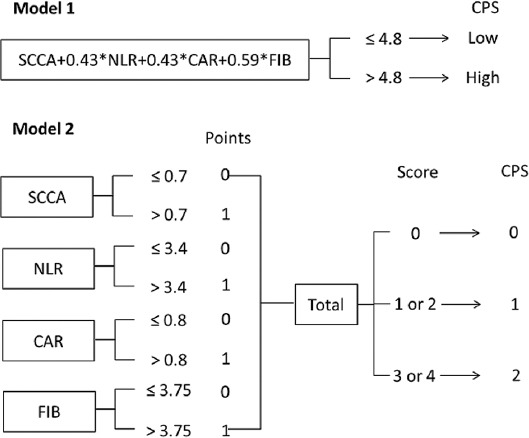
Calculation of the comprehensive prognostic score in two models. For model 1, according to the logistic regression equation, the continuous variable of CPS = SCCA+0.43 NLR+0.43 CAR+0.59 FIB. The cut-off value was 4.8 according to the ROC curve. For model 2, as a categorical variable, the optimal cut-off points for SCCA, NLR, CAR and FIB were calculated by ROC curves. Then patients were calculated into 3 groups. CPS: Comprehensive prognostic score, CAR: C-reactive protein to albumin ratio, NLR: Neutrophil to lymphocyte ratio, SCCA: Squamous cell carcinoma antigen, FIB: Fibrinogen, ROC: Receiver operating characteristic

**FIGURE 3:**
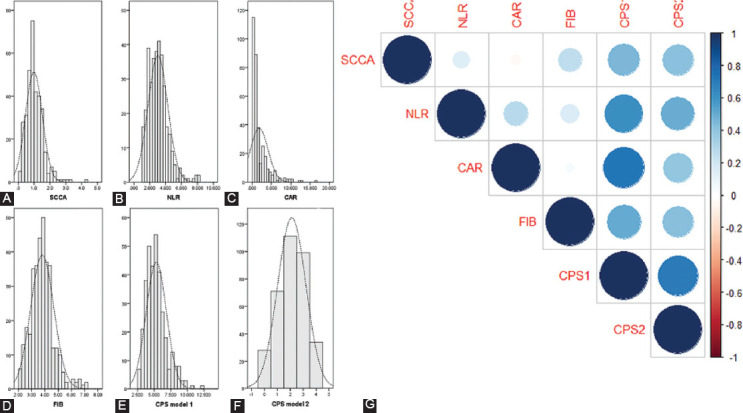
The histograms (A-F) and heatmap (G) regarding SCCA, NLR, CAR, FIB, CPS model 1 and CPS model 2. CPS: Comprehensive prognostic score, CAR: C-reactive protein to albumin ratio, NLR: Neutrophil to lymphocyte ratio, SCCA: Squamous cell carcinoma antigen, FIB: Fibrinogen

The detailed definition of CPS was shown in Figure 2. For model 1, as a continuous variable, the levels of SCCA, NLR, CAR, and FIB were firstly conducted using logistic regression analysis. Subsequently, the logistic regression equation was as follows: Y = 0.760 SCCA + 0.325 NLR + 0.328 CAR + 0.449 FIB. Thus, the continuous variable of CPS = SCCA + 0.43 NLR + 0.43 CAR + 0.59 FIB. Then, the patients were assigned to CPS model 1 by using the cut-off value of 4.8 according to the ROC curve, and categorized into 2 groups (low group: ≤4.8 and high group: >4.8, respectively). For model 2, as a categorical variable, the levels of SCCA, NLR, CAR and FIB were calculated by ROC curves to select optimal cut-off values. Then patients were calculated into 3 groups (CPS0, CPS1 and CPS2, respectively). The histograms and correlation diagrams regarding SCCA, NLR, CAR, FIB and CPS were shown in Figure 3. The comparisons grouped by CPS were shown in Table 2.

**TABLE 2:**
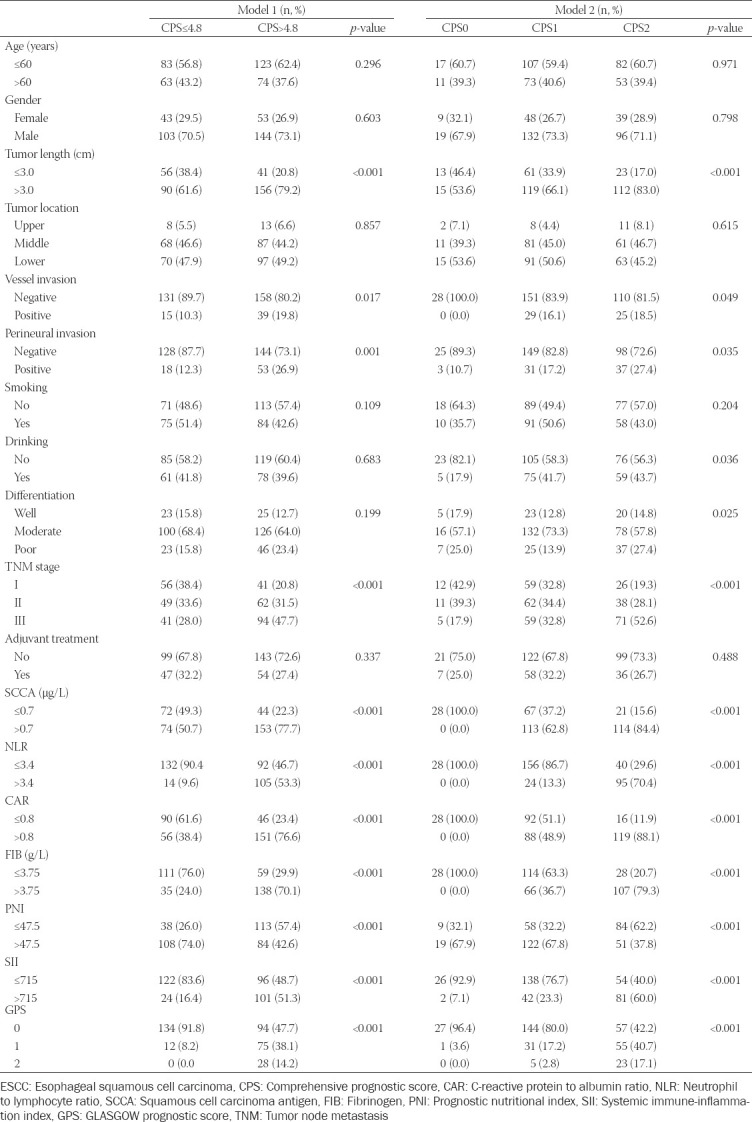
Comparison of baseline clinical characteristics based on CPS in ESCC

### AUC comparisons between CPS and other variables

**FIGURE 4:**
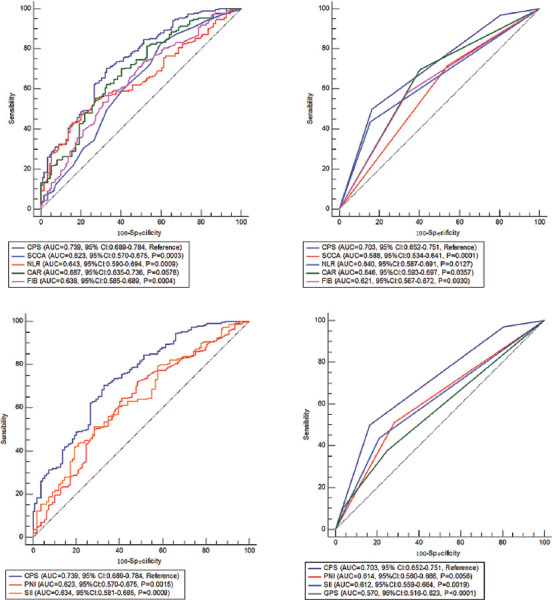
ROC analyses. The comparisons of ROC curves are continuous and categorical between CPS and its components of SCCA, NLR, CAR and FIB and other conventional prognostic scores of PNI, SII and GPS. CPS: Comprehensive prognostic score, CAR: C-reactive protein to albumin ratio, NLR: Neutrophil to lymphocyte ratio, SCCA: Squamous cell carcinoma antigen, FIB: Fibrinogen, PNI: Prognostic nutritional index, SII: Systemic immune-inflammation index, GPS: GLASGOW prognostic score, ROC: Receiver operating characteristic

The AUC values comparisons according to the ROC curves between CPS in continuous or categorical status and other variables were shown in Figure 4. The AUC values regarding CPS were 0.739 (continuous) in model 1 and 0.703 (categorical) in model 2, respectively. CPS had the largest AUC compared with either other prognostic indicators (GPS, SII and PNI) or its components (SCCA, NLR, CAR and FIB). These results indicated that higher predictive ability of CPS score on prognosis than other indicators.

### CSS analyses and subgroup analyses grouped by CPS

**FIGURE 5:**
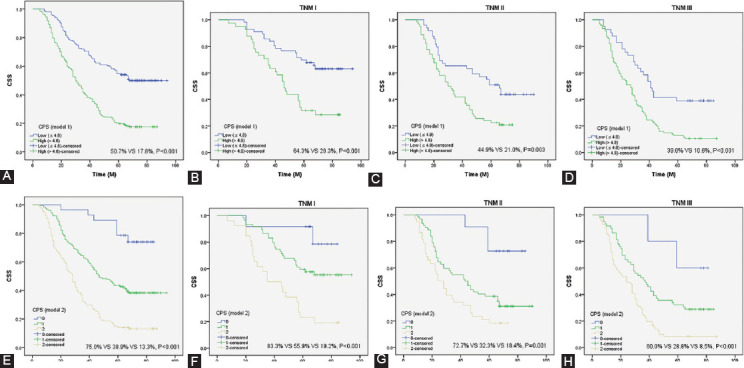
Figure legends: CSS analyses. Kaplan-Meier for CSS grouped by CPS in Model 1 (A) and Model 2 (E). CSS analyses for CPS in subgroup analyses based on the TNM stage in model 1 (B-D) and model 2 (F-H), respectively. CPS: Comprehensive prognostic score, CSS: Cancer-specific survival, TNM: Tumor node metastasis

For model 1, patients in CPS low group had the better 5-year CSS than those in CPS high group (50.7% vs. 17.8%, *p* < 0.001, Figure 5A). For model 2, the 5-year CSS for patients in CPS 0, 1 and 2 were 75.0%, 38.9% and 13.3%, respectively (*p* < 0.001, Figure 5B). To better explore the prognostic value of CPS (model 1: Figure 5C-E; model 2: Figure 5F-H), subgroup analyses based on different TNM stages were performed. These findings suggested that CPS (model 1 or model 2) had reliable abilities to predict prognosis in resected ESCC patients in any TNM stages.

### Univariate and multivariate analyses for prognostic factors

**FIGURE 6:**
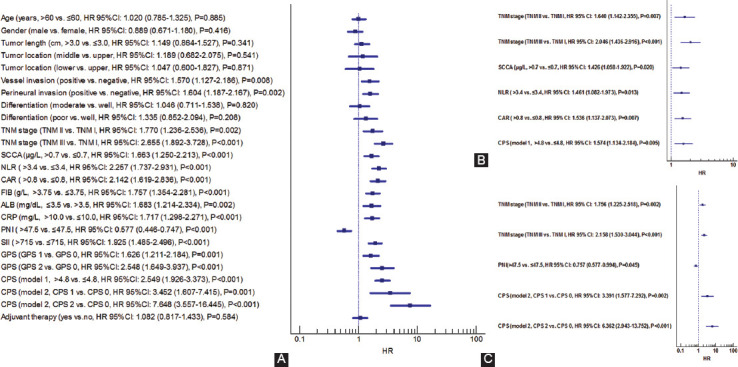
Univariate (A) and multivariate analyses for CSS regarding CPS model 1 (B) and CPS model 2 (C). CPS was an independent marker regarding CSS in resected ESCC in model 1 (HR=1.574, 95% CI: 1.134-2.184, *p*=0.005) or model 2 (CPS 1 vs. NPS 0: HR=3.391, 95% CI: 1.577-7.292, *p*=0.002; CPS 2 vs. CPS 0: HR=6.362, 95% CI: 2.943-13.752, *p*<0.001). CPS: Comprehensive prognostic score, CSS: Cancer-specific survival, ESCC: Esophageal squamous cell carcinoma

The results revealed that CPS in model 1 (*p* < 0.001) or model 2 (*p* < 0.001) was significantly associated with CSS according to the univariate analyses (Figure 6A). CPS and other significant prognostic factors were recruited in further multivariate analyses. The results in the training cohort demonstrated that CPS was confirmed as an independent score in both two models (Figure 6b and C).

### Development and validation of the nomogram

**FIGURE 7:**
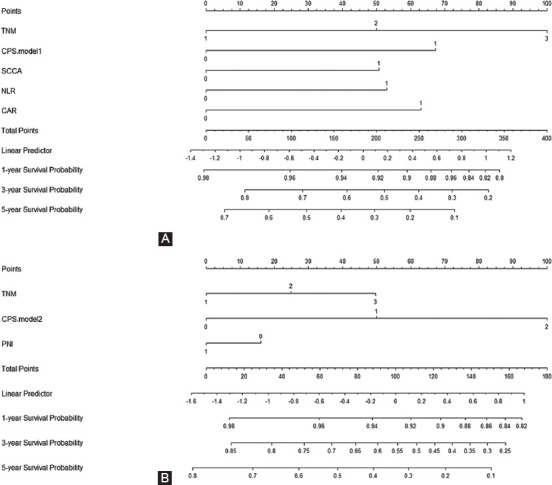
Nomogram based on CPS for predicting the 1-, 3- and 5-year CSS in model 1 (A) and model 2 (B). CPS: Comprehensive prognostic score, CSS: Cancer-specific survival

**FIGURE 8:**
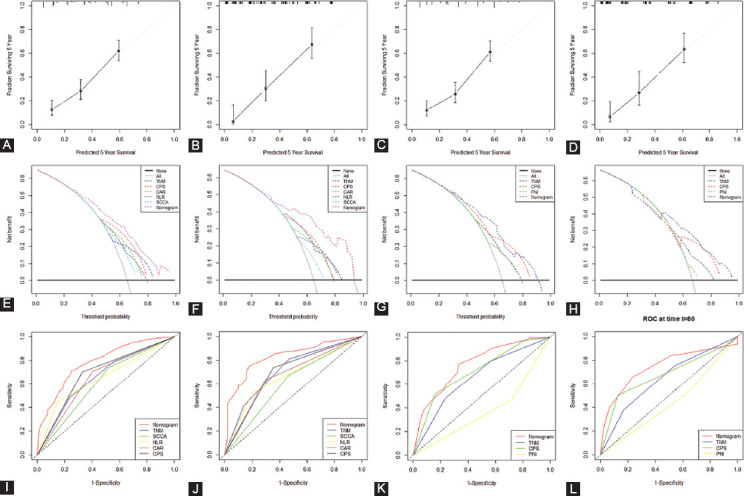
Calibration curves (A-D), decision curves (E-H) and time-dependent ROC curves (I-L) of the nomogram. Calibration curves presented an acceptable agreement between the two cohorts. Decision curve analyses revealed that nomogram models had higher overall net benefits than TNM stage. Time-dependent ROC curve analyses revealed survival prediction was significantly higher in nomogram than TNM stage. ROC: Receiver operating characteristic, TNM: Tumor node metastasis

According to the multivariate analyses, five variables (SCCA, NLR, CAR, TNM and CPS) in model 1 and three variables (CPS, TNM and PNI) in model 2 were recruited to establish two nomograms to predict individual survival (Figure 7A and B). The C-indexes were 0.689 and 0.737 in model 1 and 0.684 and 0.719 in model 2 in the two cohorts, respectively. An acceptable agreement between these two cohorts regarding the individual 5-year CSS prediction based on the calibration curves (Figure 8A-D). The CPS-based nomogram models in these two cohorts had higher overall net benefits than TNM stages according to the decision curve analysis (Figure 8E-H) and time-dependent ROC curve analyses (Figure 8I-L) regarding 5-year CSS prediction. The results confirmed that the CPS-based (either in continuous or categorical) nomogram can accurately and effectively predict survival in resected ESCC in two models.

## DISCUSSION

To date, it is a dilemma to identify which patient of ESCC will suffer an aggressive behavior with poor prognosis and whether he or she will benefit from surgical resection. Therefore, exploring more novel prognostic scores in ESCC is still an important task. The present study explored an integrative prognostic score of CPS to predict clinical outcomes and prognosis in resected ESCC patients. CPS had the largest AUC in both two models, compared with other prognostic indicators, which indicated that higher predictive ability of CPS on prognosis than other prognostic indicators and confirmed as a useful independent prognostic score. Moreover, a predictive CPS-based nomogram was established in the training cohort and validated in the validation cohort. The CPS-based nomogram can accurately and effectively predict survival in resected ESCC.

Current NCCN guidelines recommend neoadjuvant chemotherapy or chemoradiotherapy for patients with locally advanced EC with the key evidence mainly from trials in Western countries [24]. Due to the increasing differences regarding the pathological types of EC between the East (dominant SCC) and West (dominant adenocarcinoma), most patients included in the trials are inconsistent with those in Asian countries (including China) [25]. Studies have also revealed that neoadjuvant therapy may increase the risk of peri-operative mortality or post-operative morbidity for ESCC patients [26]. Therefore, a significant number of locally advanced ESCC patients in China tended not to meet the NCCN guidelines and prefer surgery as the initial treatment [27,28]. In the current study, patients with pre-operative neoadjuvant treatments were excluded because neoadjuvant treatment might affect the hematological indicators. Recent accumulating evidence indicates that patients after radical resection without neoadjuvant therapy with T3-T4 tumors and those with positive node metastasis should receive post-operative chemotherapy or post-operative chemoradiotherapy [20,21]. However, not all patients in China received post-operative adjuvant therapy, which is mainly based on the surgeon’s recommendation and the physical and financial status of each patient [18,19].

Tumor biomarkers play important significance in cancer diagnosis and prognosis and may become novel therapeutic targets. Serum SCCA, as a serine/cysteine protease inhibitor, may be involved in the malignant behavior of SCC and play an important role in cancer invasion and metastasis [29]. With regard to the prognostic value of SCCA in patients with EC, a study including 309 patients with ESCC was performed [8]. The results demonstrated that both the concentration and positivity rate of SCCA were significantly elevated in patients associated with tumor progression, suggesting that SCCA was associated with prognosis in patients with ESCC. The similar results were also confirmed in another study which indicated that serum SCCA was associated with lymph node metastasis and depth of tumor invasion [9]. However, a meta-analysis including 5 studies indicated that SCCA was not associated with survival in EC [30]. The SCCA was an independent marker in model 1 with the cut-off value of 0.8 μg/L.

Nutrition and inflammation are associated with tumor prognosis. NLR and CAR were the most widely recognized indicators for prediction of prognosis in various cancers, including ESCC [10,12]. Two meta-analyses published in recent years have found that NLR and CAR were related to prognosis in patients with EC [31,32]. In addition, some studies have reported the prognostic value of the combination use of NLR and/or CAR with other potential markers [10,11]. In the current study, NLR and CAR were independent prognostic factors in model 1. Various studies also indicated that other conventional nutrition- and/or inflammation-related indexes including GPS, PNI and SII were associated with prognosis in cancers [22,23]. CPS in the present study had the highest abilities to predict prognosis in resected ESCC compared with the common indicators of PNI, GPS and SII in ROC analyses or Cox analyses.

More and more studies in recent years have reported that high levels of plasma FIB are significantly correlated with poor prognosis in a variety of cancers, including ESCC [14,15]. Recently, plasma FIB was confirmed as a prognostic indicator in patients with EC according to two meta-analyses [33,34]. However, an opposite result was found in the current study. The results revealed that FIB was not related to prognosis in ESCC. The exact mechanism between serum FIB and cancer prognosis remains unclear. However, there are some potential explanations. The coagulation system is often abnormally activated in cancer patients, and serum FIB could possess anti-cancer properties [35,36]. In addition, serum FIB, as an extracellular matrix protein, could regulate tumor cell growth by binding to a variety of growth factors and enhance cell migration, invasion and metastasis [37].

Recently, several studies have reported that nomogram is a better method to predict prognosis in a variety of cancers [38,39]. In the current study, our nomogram based on CPS contained five variables (SCCA, NLR, CAR, TNM, and CPS) in model 1 and three variables (CPS, TNM, and PNI) in model 2. The AJCC TNM classification is the most widely used staging system for various cancers. At present, post-operative treatment and prognosis prediction for ESCC patients are mainly based on the TNM system. The CPS-based nomogram showed better discrimination than the TNM staging system. In addition, the CPS alone can predict survival better than the TNM staging system. There may be several reasons. On the one hand, several important prognostic factors, such as differentiation, lymph node sites and number of examined lymph nodes, are not included in the TNM system for ESCC [40]. Therefore, ESCC patients receiving similar therapy tend to have different prognosis at the same TNM stage, suggesting that the current AJCC TNM system, which only assesses anatomical factors, may not be sufficient to make prognosis predictions and treatment decisions. On the other hand, the potential synergy and complex interaction for the CPS used in this study including inflammatory and nutritional status, coagulation indicator and tumor marker in the tumor microenvironment, reflecting a better prognosis prediction. Two nomograms regarding CPS in two models and other variables were established. Oncologists could use these nomograms to predict individual survival prediction in daily work. The simply and easily obtained variables in nomogram, improves the application in daily clinical practice.

The cancer prognosis is related to many factors, such as nutritional and/or inflammatory status, coagulation-related indicators, and tumor markers. However, these prognostic factors may be influenced by a variety of non-cancer related conditions, which may lead to biased results. We hypothesized that the combination of these indicators could reduce the potential bias and improve the prognostic value. The present study explored an integrative prognostic score of CPS to predict clinical outcomes and prognosis in resected ESCC. Compared with other prognostic scores in the present study, CPS was confirmed as a useful independent prognostic score according to the Cox analyses. Compared to previous studies, this study had the following advantages: Firstly, most previous studies reported a single indicator or a combined indicator under the same status to predict prognosis of ESCC. To increase prognostic accuracy and reduce the potential bias, we evaluated many potential prognostic scores under different status to establish a multivariate prognostic model. Secondly, the prognostic nomogram model based on the combination of inflammatory and nutritional score, coagulation indicator and tumor marker with TNM stage system was more accurate in predicting survival than that of the conventional TNM stage system. Thirdly, our model offers a convenient method in two models to predict outcomes for surgical patients in ESCC, which provides a more personalized approach to cancer treatment in clinical practice.

Some limitations should be acknowledged. Firstly, due to retrospective character in single-center, it was correlated to certain bias and inaccuracy. Secondly, although the strict inclusion and exclusion criteria were adopted and the combination of these indicators was performed, levels of these serum variables may be affected by other conditions, the applications should be limited. Thirdly, although the validation cohort was validated by the nomogram, we also lack an additional independent external validation cohort to validate. Although the above-mentioned limitations existed, our prognostic nomogram model might serve as a useful tool for clinicians to estimate individualized survival prediction for resectable ESCC patients.

## CONCLUSION

The CPS is a novel, simple and effective predictor in resectable ESCC. The CPS has potential independent prognostic value in predicting CSS, which can accurately and effectively predict individual survival for resectable ESCC. The simply and easily obtained variables in nomogram, improves the application in daily clinical practice. The CPS may allow for treatment stratification, thereby helping clinicians provide a more personalized approach to cancer treatment.
